# Assessment of sexual and reproductive health knowledge and awareness among single unmarried women living in Lebanon: a cross-sectional study

**DOI:** 10.1186/s12978-021-01079-x

**Published:** 2021-01-28

**Authors:** Maya Hamdanieh, Louna Ftouni, Bara’a Al Jardali, Racha Ftouni, Chaymaa Rawas, Marina Ghotmi, Mohammad Hussein El Zein, Sara Ghazi, Salah Malas

**Affiliations:** Faculty of Medicine, Beirut Arab University, Beirut, Lebanon

**Keywords:** Sexual, Reproductive, Knowledge, Health, Awareness, Unmarried women, Lebanon

## Abstract

**Background:**

Sexual and reproductive health (SRH), a globally recognized fundamental health concern and a basic human right is poorly addressed and seldom researched in the Arab world. Disregarding this aspect of health creates various obstacles to accessing SRH related services and education. This threatens the health of a female, namely through increasing the probability of unplanned pregnancies and unsafe abortions, augmenting the risk of acquiring sexually transmitted infections, and most importantly, increasing the hazard of maternal and neonatal death. Thus, this study aimed to assess the level of SRH related knowledge and awareness among single unmarried women living in Lebanon.

**Methods:**

A descriptive cross-sectional study design was conducted using a self-administered questionnaire in both English and Arabic languages. The questionnaire included 9 sections; socio-demographic characteristics section, sexually transmitted infections (STIs) section, premarital tests section, vaccines section, menstruation and its abnormalities section, pregnancy symptoms and identification section, methods of contraception section, vitamins section, and honeymoon events section.

The questionnaire was distributed among all Lebanese governorates to 491 single unmarried women living in Lebanon aged between 17 and 55 years. Student t-test and Chi-Square test were used to analyze results.

**Results:**

It was found that only 8.8% of all the participants had adequate knowledge. The highest level of SRH related knowledge was about pregnancy (88.0%), and the least was about contraception (13.5%). Most of the knowledgeable participants lived in Beirut governorate (13.6%, n = 8) and had reached universities (10.3%, n = 41), but this was not statistically significant (*p*-value > 0.05). The effect of a prior visit to a gynecologist was statistically non-significant on the overall level of knowledge (*p*-value = 0.269).

**Conclusion:**

Due to the inadequate level of knowledge, SRH education campaigns empowered by the Ministry of Public Health in collaboration with primary care physicians and gynecologists, are recommended in both the societal and academic sectors to enhance the awareness level and make SRH knowledge readily available among unmarried women aged between 17 and 55. Knowing the massive role of social media nowadays, the messages they present should contribute to enhancing the level of SRH knowledge and redirect attitudes and behaviors of unmarried females in Lebanon.

## Plain english summary

Sexual and reproductive health (SRH) is an important aspect of health and a fundamental human right. It represents “a state of complete physical, mental, and social well-being in all matters relating to the reproductive system. It implies that people are able to have a satisfying and safe sex life, the capability to reproduce, and the freedom to decide if, when, and how often to do so”, as stated by the World Health Organization.

In this study, single unmarried women between the age of 17 and 55 living in Lebanon were asked about different SRH matters: sexually transmitted infections (STIs), premarital tests, certain vaccines, menstrual cycle and its abnormalities, pregnancy symptoms and identification, methods of birth control, vitamins, and honeymoon events, to assess their level of SRH knowledge. Respondents were asked to fill questionnaires and were provided with an explanation for their inquiries after the collection of the questionnaires. Of the 491 respondents, only 8.8% had an overall knowledge regarding SRH matters. The highest level of SRH related knowledge was about pregnancy (88.0%), and the least was about contraception (13.5%). The deficient SRH awareness makes females more vulnerable to poor outcomes such as high-risk sexual behaviors, deficient usage of birth control methods, the spread of STIs, sexual abuse, unwanted pregnancies, and unsafe abortions.

In conclusion, due to the lack of knowledge about SRH, education and awareness campaigns directed by specialists in this field are recommended.

## Background

Sexual and reproductive health (SRH) are two of the numerous aspects of public health that were brought to light in 1994, at the International Conference on Population and Development (ICPD) in Egypt [1]. While sexual health represents a state of physical, emotional, mental, and social well-being in relation to sexuality [2], reproductive health addresses reproductive processes, functions and system at all stages of life, and the capability to reproduce and the freedom to decide if, when, and how often to do so [3]. SRH is a broad term that encompasses a wide array of health concerns as sexually transmitted infections (STIs), premarital tests, vaccines, menstrual cycle and its abnormalities, pregnancy symptoms and identification, methods of contraception, vitamins, and honeymoon events [4, 5]. Due to the sensitivity of these issues, young individuals are provided with inadequate and misleading information on reproductive health [6]. Furthermore, cultural taboos hold back the youth in many developing countries from discussing sexual issues explicitly with their parents [7, 8]. This dearth of knowledge about SRH may have serious outcomes [8]. For instance, the lack of sexual education means that women do not have access to contraceptive methods and will be subject to unplanned pregnancies and unsafe abortions, and sexually transmitted infections [9]. Additionally, consanguineous marriages and the lack of premarital screening increases the probability of fetal or neonatal death and the long-term sequelae on the child’s health and his/her mother’s well-being [10]. However, there are other significant issues to consider, because sexual and reproductive health education poverty means that women, especially young ones, are vulnerable to sexual violence and nonvoluntary sexual encounters with all their detrimental effects on the life of those affected [11]. Thus, sexual and reproductive health education should go hand in hand with formal academic education to empower women and liberate them from the barriers and taboos forged by patriarchal societies and which threaten their lives with the aforementioned health hazards.

In comparison to Western countries in which sexual and reproductive health education is evident [12], studies in Arab countries have shown a significant lack of knowledge in premarital tests [13–15], STIs [16], contraceptives [17], and other reproductive health issues [18, 19]. In Lebanon, research on SRH matters is lacking, and no research neither locally nor internationally simultaneously approached all the issues mentioned previously*.* It was postulated that women living in Lebanon have little comprehension of SRH. Thus, the primary objective of this study was to assess SRH knowledge and awareness among single unmarried females in Lebanon who are in their reproductive years. The target was this category of women who are still not married and have supposedly not been engaged in any sexual experience with the intent to educate them and raise awareness about neglected aspects of their personal lives and health. Our secondary objectives were to discern whether proper awareness and education in those fundamental topics are being implemented in the Lebanese educational institutions such as schools and universities and to detect the geographical areas where knowledge is least satisfactory, thus, permitting to shed the light on these areas and to make them hubs for educational campaigns and programs focusing on SRH promotion, disease prevention, and policies reinforcement.

## Methodology

### Study design and setting

A cross-sectional study was designed to assess the SRH knowledge among unmarried women aged between 17 and 55 years living in Lebanon among all six Lebanese governorates. Participants were chosen by convenience sampling. Convenience sampling is a type of non-probability sampling and relies on data collection from an available pool of respondents. In other words, all women in Lebanon were invited to participate in the study, if they met the inclusion criteria.

### Inclusion and exclusion criteria

All single unmarried women living in Lebanon were eligible to participate in the study.

Many women in Lebanon gain SRH knowledge shortly before or after their marriage. The study was intended to highlight the importance of gaining this knowledge beforehand to prevent different health issues, hence unmarried women instead of married ones were targeted to assess their level of knowledge. Moreover, asking a married woman about honeymoon events, premarital vaccines, methods of contraception, and premarital tests would have shown biased results due to the already acquired knowledge from her marital experience. Thus, the target population was women aged between 17 and 55 years who are in their reproductive years. Women aged below 17 years were excluded due to access restriction to schools and the need for parental consent. Menopausal women were excluded too since they do not fall in the reproductive age group. As well, women who do not speak English or Arabic were excluded. Those who were divorced or engaged were also eliminated because they supposedly gained prior knowledge about SRH.

### Sample size and population

The sample size was determined using an online sample size calculator [20]. The calculator provided the sample size required to estimate prevalence with a specified level of confidence and precision along with the population size. Thus, for approximately a 7 million population size [20], a precision of ± 5%, and a confidence level of 95%, a representative sample of the population was generated at n = 385 [21]. Due to the sensitivity of the topic, the non-response rate was estimated at approximately 25%; consequently, a total of 491 participants constituted the study sample. By referring to the statistical bulletin of the Ministry of Public Health (2016) [22], and to represent all six governorates proportionately in the sample size, the following percentages were used: Beirut: 9.35%, Bekaa: 14.26%, Mount Lebanon: 35.6%, Nabatiyeh: 7.57%, North: 21.56%, South: 11.66%.

### Study tool and questionnaire content

Eligible participants were asked to fill a structured, self-administered questionnaire. The questionnaire was created by the authors following a thorough literature review using reliable sources such as the Centers for Disease Control and Prevention website and UpToDate [23–32]. Some questions were adapted from a WHO instrument [33]. The instrument is a questionnaire that aimed to study the SRH of young people who have not yet been married. The statements were taken specifically from Sects. 2, 7, 8, and 11 of the instrument and covered questions about the sources of information on, and knowledge about reproductive health, knowledge and ever-use of contraceptive methods, knowledge about Human Immunodeficiency Virus and Acquired Immunodeficiency Syndrome (HIV/AIDS) and sexually transmitted infections, and the use and perceptions of health services, respectively.

Other statements in the STIs section were adapted from a study assessing knowledge about STIs [34]. These statements were adjusted to fit into the context of Lebanese culture and norms.

Additionally, the questionnaire consisted of 33 closed-ended questions and 5 tables. It included 9 sections. The socio-demographic characteristics section included questions about the age, nationality, and educational level of the participant, and about the governorate of residence (i.e. the geographical area in which the participant lived). The knowledge on STIs section included questions about HIV, Human Papilloma Virus (HPV), Gonorrhea, Chlamydia, Syphilis, and Pelvic Inflammatory Disease (PID).The premarital tests section comprised of questions about sickle cell disease, blood group compatibility between couples, and some premarital screening tests like hemophilia and diabetes. The vaccines section involved questions about HPV, tetanus, hepatitis B virus, chickenpox virus, Measles-Mumps-Rubella (MMR) viruses, and influenza virus. The menstruation and its abnormalities section was comprised of questions about any previous gynecologist visit, menstrual cycle, ovulation, amenorrhea, and dysmenorrhea. The pregnancy section consisted of questions about pregnancy symptoms and detection, and about the type of food eaten by a pregnant woman in relation to the sex of the baby. Knowledge about contraception included questions about methods of birth control such as combined oral contraceptive pills (COCPs), progesterone-only pills, patches, and intrauterine devices (IUD). The vitamins section tested the participants’ knowledge with regards to folic acid, vitamin A, and vitamin D. Lastly, the honeymoon events section encompassed questions about the hymen, virginity, causes of burning sensation and prolonged bleeding and pain after the first intercourse, condoms, causes of honeymoon cystitis and ways to prevent it.

### Data collection

Data collection was carried out between July 2017 and January 2018. The authors collected the questionnaires in soft and hard copies. Online Google Forms on a digital tablet were made available to encourage participation in public places as shopping centers, large events, universities, among others. Before questionnaires’ administration, the aim of the study was explained to the participants and written consents were signed by them. After completion of the questionnaire, correct answers were discussed with the participants, and they were welcome to ask for further explanation. Five questionnaires were excluded because they were less than 80% complete, or the form was submitted twice online, or for other minor reasons. Moreover, missing data were not included in the results.

### Scoring system

Scoring was done as follows: 1 point for each correct answer and 0 points for each incorrect answer and “I don’t know” answer. Regarding the score of each section: the first section (the sociodemographics section) was excluded from the scoring system since it consisted of qualitative data. The total score for correct answers in the second section (knowledge on STIs section) was 11, in the third section (the premarital tests section) was 4, in the forth section (the vaccines section) was 9, in the fifth section (menstruation and its abnormalities section) was 12, in the sixth section (pregnancy symptoms and identification section) was 5, in the seventh section (contraceptives section) was 5, in the eighth section (the vitamins section) was 4, in the final section (the honeymoon events) was 8. Thus, the total number of correct answers was 58. Furthermore, it should be noted that one table not assessing knowledge was omitted from the scoring system. A new index was generated to estimate the overall knowledge by summing the scores of the eight sections. Knowledge was classified as “adequate” if there were 39 or more (approximately 2/3 or 67.2%) correct responses to all knowledge related questions and tables [35], and “inadequate” if the score was less than 39.

### Statistical methods

Data analysis was performed using International Business Machines Statistical Package for the Social Sciences (IBM SPSS) version 23.1. Data entry was carried out by all authors, and data entry accuracy was confirmed by cross-checking and proofreading the entered data by two of the investigators. Categorical variables were summarized into frequencies and percentages and compared using the Chi-square test. Continuous variables were presented as the standard deviation (SD) and means. The means of quantitative variables were compared using the independent t-test (Student's t-test). All tests are two-sided. A *p*-value ≤ 0.05 was considered to be statistically significant.

### Ethical considerations

A formal proposal was written to the Institutional Review Board (IRB) of Beirut Arab University which approved proceeding with the study under the following approval code 2017H-0066-M-R-0234. Written informed consent was obtained from the participants for the publication of this study. The participation was voluntary, and the respondents were informed that they could withdraw from the study at any time if they desired to do so without any penalty. Where convenient to the participant, Google Forms online platform was used to fill the questionnaire, and such forms guaranteed the participants anonymity and confidentiality by keeping each submission completely untraceable. The researchers ensured the security of hard copies and assigned a code to each questionnaire.

## Results

### General characteristics of respondents

The number of distributed questionnaires was 625 with a response rate of 78.6%. All the respondents (n = 491) were single unmarried females, and their ages ranged between 17 and 55 years, with a mean age of 22.5 ± 5.4. There was a preponderance of educated participants, the greater proportion of whom attaining a university degree (81.7%, n = 397) (Table 1).

**Table 1:** Socio-demographic characteristics of participants (N = 491)

	Total (N = 491) N (%)
Nationality^a^	
Lebanese	442 (92.1%)
Other nationalities	38 (7.9%)
Educational level^a^	
Uneducated	5 (1.0%)
Primary	14 (2.9%)
Secondary	65 (13.4%)
University	397 (81.7%)
Other	5 (1.0%)
Governorate	
Nabatiyeh	41 (8.4%)
Beirut	59 (12.0%)
Bekaa	65 (13.2%)
South	66 (13.4%)
North	123 (25.1%)
Mount Lebanon	137 (27.9%)

^a^Missing values are not shown in the table

Out of all the respondents, only 8.8% of them had 2/3 or more correct answers in the questionnaire and thus were considered to have an overall knowledge in SRH matters. It was noticeable that the participants were most knowledgeable about pregnancy symptoms and identification, and least informed about the contraception methods. Another observation would be that participants who attained university had more knowledge (10.3%) than those with a lesser educational level (Table 2).

**Table 2:** Association between the overall sexual and reproductive knowledge of unmarried females and certain socio-demographic characteristics (N = 491)

	Participant had adequate knowledge (N = 491) N (%)	*p*-value**
Age†		
Valid answers	42	0.61
Mean ± SD^ß^	23.0 ± 3.7	
Educational level*		
Uneducated	0 (0.0%)	0.171
Primary	1 (7.1%)	
Secondary	1 (1.5%)	
University	41 (10.3%)	
Other	0 (0.0%)	
Governorate*		
Nabatiyeh	1 (2.4%)	0.126
Mount Lebanon	8 (5.8%)	
South	4 (6.1%)	
Bekaa	6 (9.2%)	
North	16 (13.0%)	
Beirut	8 (13.6%)	

ßSD signifies standard deviation

†Student t-test was used for the analysis of this data

*Chi-Square test was used for analysis of these data

**p-value < 0.05 is considered statistically significant

### Knowledge about sexual and reproductive health

The pregnancy section scored the highest level of knowledge among all Sects. (88.0%, n = 432) (Table 3). Expectedly, the largest percentage of respondents identified that urine and blood tests can detect pregnancy (83.9%, n = 411; 61.7%, n = 303, respectively), and that missed period and nausea are symptoms of pregnancy (92.2%, n = 452; 79.6%, n = 390, respectively). And, when asked whether or not the type of food affects the sex of the fetus, more than two-thirds (68.2%, n = 334) disapproved of this statement.

**Table 3:** Overall sexual and reproductive knowledge presented according to the eight sections (N = 491)

Sections	N = 491 N (%)
Pregnancy knowledge	432 (88.0%)
Premarital test knowledge	219 (44.6%)
Menstruation knowledge	175 (35.6%)
Honeymoon knowledge	170 (34.6%)
STI knowledge	84 (17.1%)
Vitamins knowledge^a^	75 (15.3%)
Vaccines knowledge^a^	74 (15.1%)
Contraception knowledge^a^	57 (13.5%)
Overall knowledge	43 (8.8%)

^a^Missing values are not shown in the table

Next is the premarital tests section, in which the level of knowledge exceeded 50% (55.4%, n = 272) (Table 3). In this section, participants were asked about the premarital tests available in Lebanon. Particularly, there were questions about the importance of sickle cell disease, blood group compatibility, and hemophilia. Additional questions as to whether the screening for diabetes mellitus and influenza virus are included in the premarital tests were also included. Most of the participants were aware of the importance of blood group testing and compatibility (66.6%, n = 327) and of hemophilia screening (71.9%, n = 351) as premarital tests. While the majority refuted the possibility of screening for influenza virus before marriage (77.9%, n = 380), less than half (42.8%, n = 209) agreed that screening for diabetes is recommended premaritally in Lebanon.

**Fig. 1:**
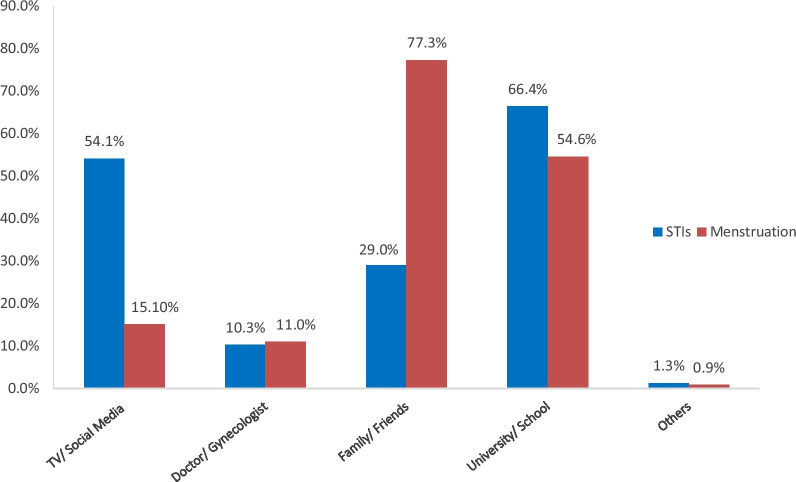
Variation in the percentage of knowledge about STIs and menstruation according to variable sources of information. (N = 445)

Concerning menstruation and menstrual abnormalities, the overall level of knowledge in this section was 35.6% (n = 175) (Table 3). Results showed that the highest sources for obtaining information about menstruation were family and friends (77.3%, n = 433) (Fig. 1). Concerning the average menstrual cycle duration, 75.1% (n = 368) of the participants answered incorrectly; and of whom 61.2% (n = 300) responded that the average menstrual cycle duration is between 4 and 10 days. Also, less than half of the participants (46.1%, n = 226) were aware of the calculation of the menstrual cycle, which extends from the first day of a period to the first day of a period of the next cycle. It was noticeable that only 47.3% (n = 232) of the participants acknowledged that ovulation occurs in the middle of the menstrual cycle. Questions about amenorrhea and dysmenorrhea revealed, on average,^1^ 49.8% correct answers. Respondents were cognizant of the proposed causes of amenorrhea in the questionnaire: pregnancy (81.8%, n = 401), hormonal dysfunction (79.0%, n = 387), stress and heavy exercise (53.7%, n = 263), and Polycystic Ovary Syndrome (PCOS) (59.1%, n = 290), except for eating disorders (e.g., anorexia) (39.5%, n = 193). Likewise, respondents were aware of the causes of dysmenorrhea suggested in the section: stressful life events (47.0%, n = 230), and ovarian cysts and tumors (61.1%, n = 299), and most of the respondents (68.1%, n = 333) recognized that dysmenorrhea can be accompanied by nausea, vomiting, and fatigue. On the other hand, a considerable number of participants (62.6%, n = 306) either answered incorrectly or did not know how to calculate the start of pregnancy, which counts from the first day of the last menstrual period.

Furthermore, the honeymoon events section comprised 9 questions related to the hymen, bleeding, pain, and burning sensation during and/or after the first intercourse, causes of pain after intercourse, types of lubricants and condoms, and honeymoon cystitis. In this specific section, 34.6% (n = 170) of the participants had adequate knowledge (Table 3). Each question in this section obtained at least 40.0% correct answers, except for two questions that had very low scores; one related to the usage of the appropriate lubricants with condoms (13.2%, n = 63), and another related to the method(s) of prevention of honeymoon cystitis (21.8%, n = 107).

In the STIs related section, questions about HIV, HPV, gonorrhea, chlamydia, syphilis, and PID revealed a knowledge level of 17.1% (n = 84) (Table 3). Out of all the participants, 92.7% (n = 454) heard about STIs, 95.4% (n = 432) of whom knew that treatment should be considered both for the affected person and their partner(s). Inquiries regarding the source of knowledge about STIs revealed that university and school were the leading sources for such knowledge (66.4%, n = 302) (Fig. 1). Despite acquiring knowledge about STIs from trusted sources, only 33.2% (n = 162) of participants were aware that HIV is transmitted by breastfeeding and 25.9% (n = 126) disapproved of the transmission of HPV through toilet seats. Additionally, only 26.1% (n = 127) of the participants acknowledged that gonorrhea causes infertility in both men and women, while 24.8% (n = 120) recognized that chlamydia can be both prevented and cured. Nevertheless, approximately half of the participants were aware of the causality between HPV and cervical cancer (50.9%, n = 248), and of the transmission of syphilis through open sores on the mouth, lips, and genitals (43.2%, n = 211). It was observed that the majority of the participants (84.4%, n = 413) did not know whether PID causes ectopic pregnancy only or infertility too.

Concerning the vitamins, a few participants were aware of them and their role in the life of a childbearing female, with only 15.3% (n = 75) scoring adequately in this section (Table 3). The percentage of participants who heard about folic acid was 59.7% (n = 290), among whom 37.5% (n = 110) hardly knew that folic acid should be administered before and in the early weeks of pregnancy, and 35.0% (n = 103) acknowledged the importance of folic acid for the growing fetus. However, it should be noted that the relation between recognizing folic acid as a vitamin and answering the questions related to its importance during pregnancy (*p*-value = 0.541), and its time of usage (*p*-value = 0.172) was not significant. Furthermore, when inquiring about the importance of vitamin D during pregnancy, the majority (71.2%, n = 346) were aware of the relation between vitamin D and the fetus’ bone and teeth health, while only 23.0% (n = 112) recognized that congenital birth defects are consequences of the excessive intake of vitamin A during pregnancy.

With respect to the vaccines section, the ones covered in the questionnaire were the HPV vaccine, the tetanus vaccine, the hepatitis B vaccine, the varicella vaccine, the MMR vaccine, and the influenza vaccine. Lack of awareness about these vaccines was detected among participants (Table 3). One important vaccine related to the premarital, as well as the marital health is the HPV vaccine, as HPV is known to cause respiratory papillomatosis in neonates born to mothers infected with the virus as well as causing certain cancers (cervical, anal, laryngeal, …). In spite of the pivotal role that the HPV vaccine plays, only 9.9% (n = 48) of participants knew that the HPV vaccine should be administered during childhood, while 38.1% (n = 185) were aware that it can be given premaritally, and merely 1.0% (n = 5) answered both questions correctly. Regarding tetanus and hepatitis B vaccines, at maximum, half of the participants acknowledged their value during childhood (48.9%, n = 235; 53.1%, n = 257, respectively). In contrast, the vast majority recognized that varicella and MMR vaccines are most frequently part of the childhood vaccination (82.5%, n = 402; 81.1%, n = 395, respectively). Likewise, nearly two-thirds of the participants (64.6%, n = 316) answered correctly with regards to the yearly vaccination against the influenza virus.

At last, the respondents were most deficient in the contraception Sect. (13.5%, n = 57) (Table 3). Yet, there was no method of contraception mentioned in the questionnaire that, at least, some of the participants had not heard of: IUD (71.3%, n = 348), COCPs (57.8%, n = 282), progesterone-only pill (mini pill) (44.1%, n = 215), and patch (24.0%, n = 117). However, no relationship existed between recognizing different methods of contraception and answering the questions related to them correctly (p-value > 0.05). It should be noted that “I don’t know” was the most frequent answer to all of the statements in this section: date of initiation of the COCPs in the menstrual cycle, type of cancer decreased by COCPs intake, relation between breastfeeding and progesterone-only pill, and function of the IUD.

A hypothesis was generated regarding the effect of a previous visit to a gynecologist on the overall level of knowledge of a participant, where it was postulated that such visits should increase the level of knowledge of any given participant.

Using Chi-Square test for associations, it was found that, while approximately one-half of the respondents (47.7%, n = 234) visited a gynecologist once in their life, only 10.3% (n = 24) of them were considered to have an overall knowledge (i.e. they had 2/3 or more correct answers in the questionnaire). This percentage was surprisingly very close to the percentage of women who did not visit a gynecologist but still had an overall knowledge (8.8%, n = 19) (Table 4).

**Table 4:** Association between a gynecologist visit and the overall level of knowledge (N = 491)

		Female had a knowledge		*p*-value**
		No N (%)	Yes N (%)	
Occurrence of a visit to a gynecologist*	Yes	210 (89.7%)	24 (10.3%)	0.269
	No	238 (91.2%)	19 (8.8%)	

*Chi-Square test was used for the analysis of this table

**p-value < 0.05 is considered statistically significant

## Discussion

This study aimed to assess the level of knowledge and awareness related to SRH among single unmarried women living in Lebanon. The overall level of knowledge among the participants was found to be 8.8%. This could be explained by the shortage of educational campaigns in schools and universities, and perhaps the neglection of this aspect of health by the Ministry of Public Health and the Ministry of Education, due to its sensitivity in the middle eastern culture and its consideration as a taboo in these societies [8, 36]. When compared to studies done in Portugal and Hong Kong [12, 36], females in Lebanon had lower levels of knowledge. This may be due to the fact that most females in the former countries were sexually active [12, 36] and had sexual education incorporated in their academic curricula, whereas such a form of education is not included in the Lebanese curricula and necessitates its implementation. Besides, the Ministry of Public Health in Lebanon offers reproductive health services but scarce sexual health services, since the last national awareness day on SRH was held in 2018 [37]. The absence of implementation of awareness regarding SRH has serious health consequences on individual and societal levels such as the transmission of STIs like HIV, sexual abuse and violence, unwanted pregnancies, unsafe abortions, and maternal and neonatal deaths [9]. When compared to other countries around the world, the findings were similar to studies done in China, Turkey, and Africa [38–40].

Moreover, it is seen that women in Beirut governorate had the highest percentage of adequate knowledge. This might be because the cultural reservation is more evident in remote governorates, compared to the capital, as noticed during the data collection phase of this study.

Despite the low percentage of overall knowledge, the majority of women had adequate knowledge regarding pregnancy which may be a product of interventions done previously all over Lebanon like Charafeddine et al. did. They made a preconception health education among schools which led to substantial improvement in the knowledge [41]. Moreover, the perception of SRH in the Middle East -due to its sensitivity- is mainly concerned with childbearing, neglecting the other aspects of SRH like vaccination [42]. These factors may have led to the discrepancy in knowledge between pregnancy and the other aspects of SRH among the participants. Nevertheless, Charafeddine et al. recommended expanding interventional educational programs regarding pregnancy to universities [41].

Moreover, the menstrual cycle constitutes a process in which female hormones stimulate an ovarian follicle to grow and release an egg. The fertilization of the egg by a sperm forms an embryo, but if left unfertilized leads to menstruation; i.e. the endometrial shedding and subsequent blood release from the vagina [43]. The menstrual cycle happens over a period of time that varies from a woman to another, averaging between 28 and 35 days [30]. More than half of the participants did not accurately recognize the average menstrual cycle duration. This lack of awareness draws attention to the participants’ confusion between the average menstrual cycle duration (28–35 days) and menstruation duration (≤ 8 days) [30]. This confusion is possibly due to the marginalization of the process of puberty [44]**,** where females stated they had limited knowledge and comprehension about menstruation before reaching menarche [45]. This shows the lack of awareness of parents, particularly the mothers who were the most common source of knowledge in several studies regarding teaching their daughters about menstruation [45–48]. The figures in the aforementioned studies were similar to those presented in this study. Such issues may not be discussed before puberty and many misconceptions may be passed on to their daughters because of the lack of sound and adequate information regarding this matter, the thing that would lead to anxiety [45] and may create a chain of generations passing misconceptions to the younger ones. Consequently, this issue needs to be corrected by authoritative sides such as healthcare workers, who are informed enough and trained [36] to deliver the right information, correct the misconceptions and fill the gaps of knowledge regarding this matter. All this could be done under the supervision of the Ministry of Public Health and the Ministry of Education. More than half of the participants acknowledged polycystic ovary syndrome (PCOS) as a cause of amenorrhea. Previous research done in Lebanon documented that PCOS was responsible for half of the cases of menstrual irregularities [49]. A probable justification for this phenomenon is that PCOS is the most common endocrine disorder among females of reproductive age affecting about 10% of women according to Rotterdam and Androgen Excess and Polycystic Ovary Syndrome (AE-PCOS) Society [50, 51], as well as it is one of the most common causes of secondary amenorrhea [52]. This high prevalence of PCOS could be a likely explanation for the promoted public awareness about such a common syndrome in the Mediterranean region. Additionally, participants who visited a gynecologist had no superior knowledge to those who did not. A study done in Lebanon in 2009 stated that women knew about sexual matters mainly from friends, schools, books, and mass media, but none of them had information from healthcare workers [16]. While the International Federation of Gynecology and Obstetrics’ (FIGO) envisions all women to achieve the highest conceivable standards of mental, physical, sexual, and reproductive health and well-being throughout their lives [53], there seems to be a defect in Lebanon, in the role of some obstetricians and gynecologists, as well as some primary care physicians, on working under this vision.

Although most of the respondents had heard about STIs from university or school, television and social media were the primary sources of adequate knowledge about STIs. The results were similar to a study done in Lebanon which revealed that mass media has a significant influence on the perceptions and opinions of the youth [16]. This could be added to the fact that schools and universities are not providing sufficient information about STIs. Furthermore, only one-third of the participants knew that HIV could be transmitted by breastfeeding, in contrast to a study conducted in the United Arab Emirates (UAE) which demonstrated some knowledge on the modes of transmission of HIV/AIDS (Acquired Immunodeficiency Syndrome). Misconceptions such as getting HIV from public toilets, mosquito bites, or from touching an HIV infected person are still present in UAE [54] and Lebanon. In opposition, British, American, and Swedish studies showed that the public had a high level of knowledge about the ways of transmission of HIV [55]. This diversity might be attributed to differences in beliefs, cultures, religions, and school curricula, all of which have a significant impact on knowledge.

Besides, the results revealed that only 1 out of 100 women acknowledged that the HPV vaccine should be given during childhood, or if not, before marriage. This lack of knowledge is possibly due to the neglect of healthcare workers in raising awareness about the value of this vaccine among women. This is in accordance with the results of studies held in Spain [56] and Pakistan [57]. The latter suggested that the false perceptions surrounding HPV vaccination could be due to a lack of knowledge, fear of adverse consequences, and the refusal of vaccinations by the healthcare professionals in Pakistan. These factors ultimately altered the perception of the vaccination by the general population and students specifically [57]. It is imperative to reinforce education about HPV [56] and to add HPV vaccination to the mandatory routine vaccines program in Lebanon [58]. This vaccine is excluded from the current vaccination program and is under-promoted at the time of increasing sexual activity and lack of awareness about potential serious sequelae. The effects of HPV such as genital warts and various types of cancer namely cervical and orogenital cancer [59], could be prevented if addressed in the right way, starting by obtaining sufficient knowledge and awareness regarding this topic.

Additionally, in the present study, results showed that most of the females had heard about folic acid. This is similar to two other studies in Lebanon and in the Middle East where females had also heard about folic acid, yet most of them didn’t know that folic acid intake prevents neural tube defects [60, 61]. Likewise, a study done in Korea showed that participants were not aware of the importance of folic acid intake before and in the early weeks of pregnancy [62]. This further necessitates raising the awareness in the public, schools, and universities about the importance and time of usage of folic acid, where it was seen that the more a woman is informed about folic acid supplementation, the more likely she is going to take it [62]. This lack of knowledge is reflected with serious consequences where the rate of neural tube defects in Lebanon is estimated at 13.2 per 10,000 live births [41] compared with 5.5 per 10,000 live births in the United States [63].

Although many participants had heard about IUD and COCPs, the results did not reflect adequate knowledge. Several studies from Lebanon, Mesoamerica, Vietnam, and other developing countries, similarly showed a low level of knowledge about contraception [18, 64–66]. In contrast, two other studies; one in Tanzania and another in Nigeria revealed the presence of knowledge among females of different age groups [67, 68]. The possible basis for this dichotomy lies in that healthcare workers are one crucial source of knowledge about contraceptives in both Tanzania and Nigeria [67, 68], but not in Lebanon and Mesoamerica where the source(s) is (are) yet to be known [18, 64].

### Points of strength

The importance of this study lies in that it focuses on SRH knowledge, the lack of which leads to increasing the prevalence of sexual violence and exploitation, STIs like HIV and HPV, unplanned pregnancies, and unsafe abortions. Also, this study may guide family planning strategies by highlighting the notion of contraception and raising the level of awareness regarding the various methods of birth control and normalizing their use. Apart from that, this is the first study targeting simultaneously various SRH related issues among single unmarried women living in Lebanon. It also provides basic concepts about sexual and reproductive knowledge on top of which effective educational campaigns and sustained programs can be designed and implemented.

### Limitations

Where this study has points of strength, it is not free of limitations. First, this is a cross-sectional study, meaning that it may reflect associations but causal relationships cannot be drawn. Second, most of the participants were in their twenties which could not provide a better overview of the knowledge across all age groups. The level of knowledge may be reflected by younger age groups who may be less informed regarding SRH. Nevertheless, these limitations did not affect the objectives of the study.

## Conclusion

This study questioned several aspects of SRH knowledge among unmarried females in Lebanon. It was found that a variable level of knowledge existed among the participants and across different sections. Pregnancy-related matters were the participants’ area of strength and highest knowledge, while contraception was their area of weakness and lowest in knowledge. Moreover, the sections about STIs, premarital tests, vaccines, menstruation, vitamins, and honeymoon events scored inadequate levels of knowledge.

Overall, only 8.8% of the participants had adequate knowledge. Consequently, it is recommended to implement SRH education campaigns for the public, as well as in the curricula of schools and universities. Besides, Lebanese policymakers namely the Ministry of Public Health and Ministry of Education and healthcare workers, in particular gynecologists and primary care physicians, need to work on making preventive methods readily available and accessible and to provide facilities for early diagnosis and treatment of symptomatic diseases so that awareness reflects as a real benefit. Similarly, due to the increasing importance that mass media has acquired, especially in the past few years, its role in offering correct information and fostering the right practices and attitudes is crucial.

The above recommendations aim to promote health concepts and minimize myths, taboos, and misconceptions revolving around sexual and reproductive life. Additional research ought to be done to assess the level of sexual and reproductive knowledge among Lebanese men and to compare Lebanese to non-Lebanese population.

## Data Availability

The datasets used and/or analyzed during the current study are available from the corresponding author on reasonable request.

## Abbreviations

AE-PCOS: Androgen Excess and Polycystic Ovary Syndrome
AIDS: Acquired Immunodeficiency Syndrome
COCPs: Combined Oral Contraceptives
FIGO: International Federation of Gynecology and Obstetrics’
HIV: Human Immunodeficiency Virus
HPV: Human Papilloma Virus
IBM: International Business Machines
ICPD: International Conference on Population and Development
IRB: Institutional Review Board
IUD: Intrauterine Device
MMR: Measles-Mumps-Rubella
PCOS: Polycystic Ovary Syndrome
PID: Pelvic Inflammatory Disease
SD: Standard Deviation
SPSS: Statistical Package for the Social Sciences
SRH: Sexual and Reproductive Health
STI(s): Sexually Transmitted Infections
UAE: United Arab Emirates
WHO: World Health Organization

## Footnotes

1 On average means the sum of the percentages of correct answers from all the questions in the sections of amenorrhea and dysmenorrhea divided by the total number of questions in these two sections.
